# Assessment of the use of contrast enhanced ultrasound in guiding microdissection testicular sperm extraction in nonobstructive azoospermia

**DOI:** 10.1186/s12894-018-0367-y

**Published:** 2018-05-23

**Authors:** Shijun Zhang, Jing Du, Ruhui Tian, Shaowei Xie, Fenghua Li, Zheng Li

**Affiliations:** 1 Department of ultrasound, Renji Hospital, School of Medicine, Shanghai Jiaotong University, 160 Pujian RD, Shanghai, 200127 China; 2 Department of Andrology, Shanghai General Hospital, Shanghai Jiaotong University, 100 Haining RD, Shanghai, 200080 China

**Keywords:** Contrast-enhanced ultrasound, Microdissection testicular sperm extraction, Non-obstructive azoospermia, The sperm retrieval rate, Johnson score

## Abstract

**Background:**

The aim of this study is to assess the value of contrast-enhanced ultrasound (CEUS) as a new non-invasive approach to locate the testicular area in which spermatogenesis is most likely to be found in non-obstructive azoospermic testes and to evaluate the accuracy of CEUS as a predictor of successful sperm retrieval.

**Methods:**

CEUS was performed in 120 nonobstructive azoospermia (NOA) patients. Microdissection testicular sperm extraction (M-TESE) was performed on the best and poorest perfusion areas selected by CEUS and on conventional areas.

**Results:**

In the 187 testicles that underwent M-TESE, the sperm retrieval rates (SRRs) in the best perfusion area and poorest perfusion area over the maximal longitudinal section and conventional area were 63.1, 34.7 and 47.1%. According to receiver operating characteristic (ROC) analysis, the arrival times (AT) ≤27 s, time-to-peak intensity (TTP) ≤45 s, and peak intensity (PI) ≥11 dB were the best predictors of positive sperm retrieval. The location of the best perfusion area was able to guide M-TESE to improve the success rates.

**Conclusions:**

Testicle CEUS is suggested to be performed in all patients with NOA. If AT≤27 s, TTP ≤ 45 s or PI≥11 dB are found in the best perfusion area, M-TESE is strongly recommended.

## Background

Microdissection testicular sperm extraction (M-TESE) combined with intracytoplasmic sperm injection (ICSI) has become a successful treatment strategy for patients with non-obstructive azoospermia (NOA). However, under certain conditions, sperm retrieval may be challenging. The sperm retrieval success rate for patients with NOA ranges from 42 to 62% and seems to be related to the method of retrieval, patient selection or histological patterns [1–3]. NOA is caused by testicular failure, which can be attributed to genetic disorders (such as sexual chromosomal abnormalities, translocation and microdeletions of the Y chromosome), cryptorchidism, testicular torsion, radiation and toxins [4, 5]. It is well established that isolated regions of spermatogenic tissue may exist in the testes of men with NOA [6, 7]. Some studies have suggested a correlation between testicular perfusion and focal spermatogenesis [8, 9]. Testicular sperm extraction has been improved by the guidance of Doppler ultrasound in several studies, but Doppler ultrasound could not precisely localize seminiferous tubules containing sperm [2, 8, 10–13] because it was not able to show the testicle microvasculature. In this study, the microvasculature within the testicle was depicted by contrast enhanced ultrasound (CEUS), and spermatozoa residuals were most likely to be found in hyperperfused regions of non-obstructive azoospermic testes. Therefore, our study aimed to assess the accuracy of CEUS as a non-invasive approach to guide sperm retrieval and to evaluate whether it could be used as a predictor of successful sperm retrieval.

## Methods

This prospective study was approved by the institutional review board, and written informed consent was obtained from all patients.

All the patients were collected in Renji hospital between 2015 and 2016. In the CEUS group, CEUS bilateral testis was performed in 120 consecutive patients with NOA. The control group was collected from the NOA patients, retrospectively, in 2015, and they did not receive CEUS. The diagnosis of NOA was based on the complete medical history and a physical examination, and at least three different semen specimens were analysed to confirm azoospermia according to the guidelines of WHO [14]. A relevant clinical history and examination were recorded, including age, testis volume, FSH value, and karyotype analysis. No patient had prior biopsies.

The testicle volume was calculated by the Lambert formula, length × height × width × 0.71 [15]. CEUS was performed with a PHILIPS IU22 scanner equipped with a 9 MHz L9–3 transducer, which was capable of simultaneously displaying the low mechanical index (MI) mode and a grey-scale image on a split screen. The testicles were held with a hand during CEUS to keep them from moving in the scrotum, and the epididymis was kept to the backside of the testicles. Under these conditions, the testicle was in a fixed position, which was essential to locate the best perfusion area for M-TESE. The maximal longitudinal section of the testicle was selected for CEUS, and the MI for CEUS was 0.06; then, a dose of 2.4 ml of sulfur hexafluoride microbubbles (SonoVue, Bracco, Milan, Italy) was injected into a cubital vein in a bolus via a 20-gauge needle, followed by a flush with 5 mL of 0.9% normal saline. CEUS clips until 150 s after the injection were recorded continuously for further quantitative analysis. CEUS was performed on the contralateral testicle in the same manner.

**Fig. 1:**
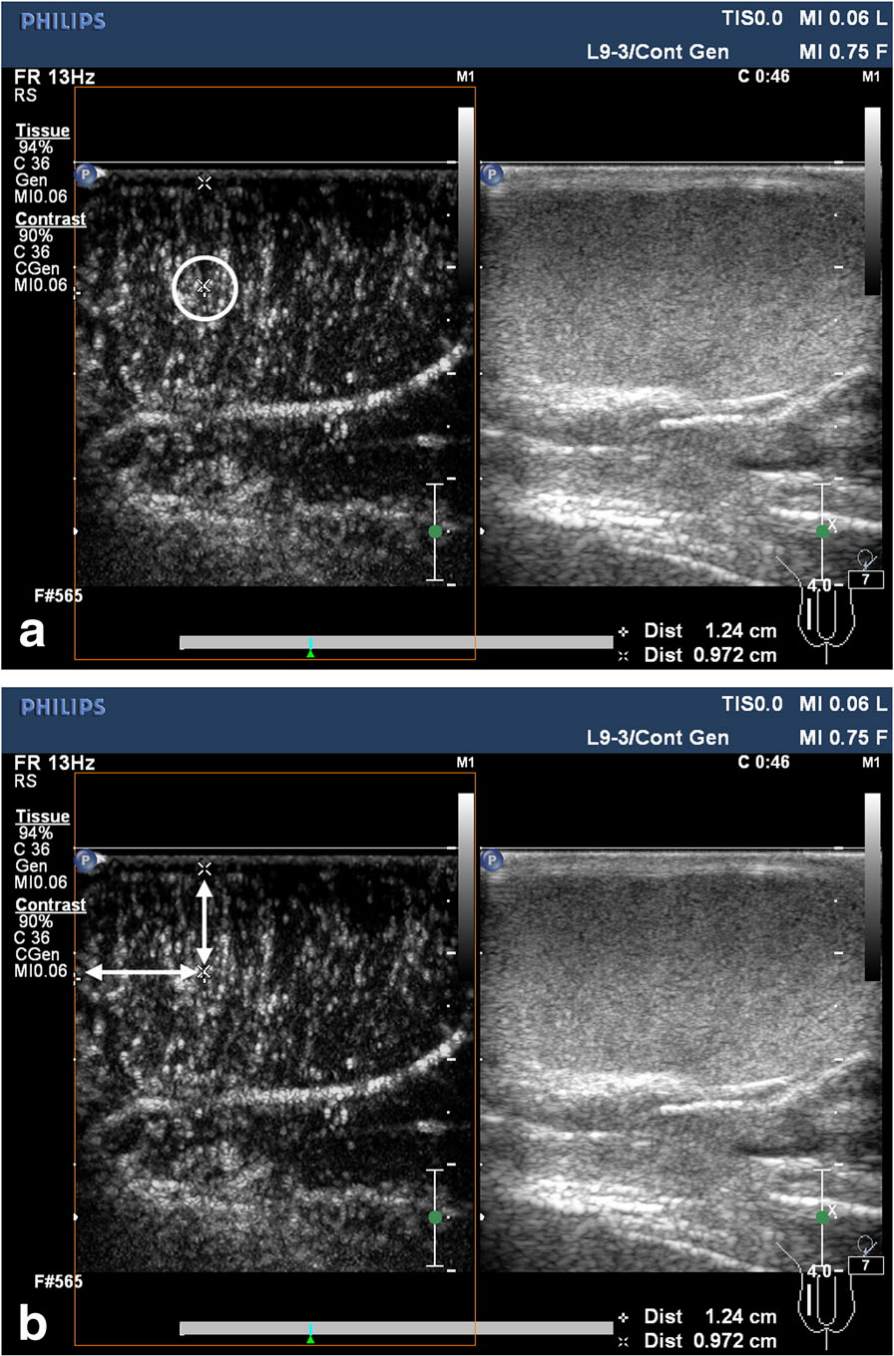
The best perfusion area analysis and location. **a**: The best perfusion area (white circle) was selected. **b**: The distance between the best perfusion area and upper pole/anterior of the testicle was measured

All images were analysed independently by two investigators (SJZ who has 11 years of experience in testes US, and SWX who has 10 years’ experience in testes US). For every testicle, the best and poorest perfusion areas over the maximal longitudinal section were chosen, and the main vessels were not included in these areas. The distance between the best/poorest perfusion area and upper pole/anterior of the testicle was measured to locate potential spermatogenesis during M-TESE (Fig. 1). Time-intensity curves (TICs) were derived from the imaging sequences, and the regions of interest (ROIs) were manually placed on the best and poorest perfusion areas on CEUS. In quantitative analysis of CEUS, the following parameters were considered:

Arrival time (AT) - the time from the injection of the agent to the point when the first contrast bubbles appeared in the testicle;

Time-to-peak intensity (TTP) - the time it took for the image intensity to reach the maximum from the injection of the agent;

Peak intensity (PI): the maximal intensity of the TIC;

Area under the curve (AUC): the area under the TIC.

The PI of the whole testicle (PIT) and AUC of the whole testicle (AUCT) were also analysed.

AT and TTP indicated the microbubble flow velocity in blood vessels. The TTP also indicated the testicle capillaries. PI indicated the maximum number of microbubbles in the capillaries, so it represented the number of capillaries. PIT indicated the number of capillaries in the entire testicle, and AUCT indicated the microbubble distribution in the entire testicle [16].

All patients underwent sperm retrieval one week after CEUS. In the CEUS group, the testicle was held in the same position as in CEUS. An incision in the equatorial plane along the mid portion at the anterior of testicle was made while the epididymis was fixed at the backside of the testicle. The mid-portion of the tunica albuginea was widely and equatorially opened in an avascular region, and with the use of an operating microscope, and seminiferous tubules that appeared larger, opaquer, and whiter were selected for seeking sperm [17]. Then, the seminiferous tubules in the best and poorest perfusion areas on CEUS were obtained separately using M-TESE. All specimens were examined by an embryologist for the surgical procedures. Any viable sperm present was cryopreserved for future ICSI. Specimens taken from the best perfusion area were sent for pathological analysis, and a Johnson score was determined for each specimen. If there was no sperm found in the testicle, the contralateral testicle underwent M-TESE in the same manner. The control group patients underwent M-TESE in the same manner without CEUS guidance. The testicle tunica albuginea was opened randomly at the upper, mid, and lower portions for seeking sperm.

Student’s t-test and the chi-square test were used to compare the SRR and CEUS parameters. The correlation between the CEUS parameters and Johnson score were analysed by correlation analysis. All statistical analyses were performed using SPSS 16.0 (SPSS Inc., Chicago, USA), with *p* < 0.05 considered to be statistically significant.

## Results

**Fig. 2:**
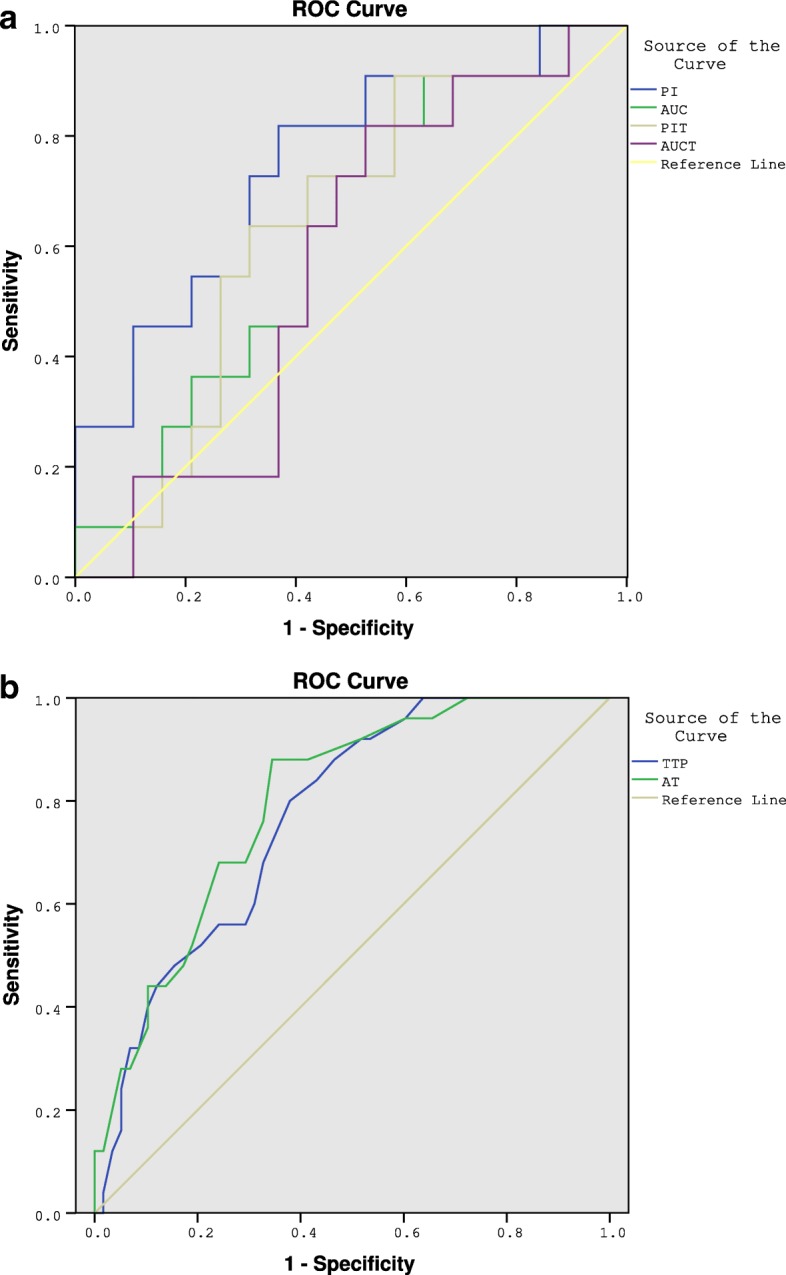
ROC curve. PI, TTP and AT were the best predictors of positive sperm retrieval. The two images respectively showed the ROC curves of PI, AUC, PIT, AUCT (a) and TTP, AT (b) in predicting positive sperm retrieval

In the CEUS group, the mean age of 120 patients was 28.3 (21–38) years, and the mean testis volume was 7.3 ml (1.8–11.6 ml). The FSH value range was 4.25 IU/L-46.43 IU/L, and the mean value was 13.49 IU/L. There were 187 testes from 120 patients who received M-TESE. Sixty-seven patients received bilateral and 53 patients received unilateral M-TESE. Sperm was retrieved from 79 testes, and the overall sperm retrieval rate was 65.8% (79/120). In the control group, sperm was retrieved from 19 patients, and the SRR was 47.5% (19/40). The SRR of the control group was lower than CEUS-guided M-TESE (*P* < 0.05). In the 187 CEUS-guided procedures, the sperm retrieval rates (SRRs) in the best perfusion area, poorest perfusion area (over the maximal longitudinal section) and conventional area were 63.1, 34.7 and 47.1%, respectively(*p* < 0.05). The correlation between the Johnson score and CEUS parameters obtained in the best perfusion area are shown in Table 1. PI and PIT had a positive correlation with the Johnson score (*P* < 0.006; *P* < 0.031). However, AT and TTP had a higher negative correlation (*P* < 0.001). AUC and AUCT had no correlation (*P* > 0.05). According to ROC analysis, AT≤27 s, TTP ≤ 45 s, and PI≥11 dB were the best cut-off point for predicting positive sperm retrieval (Fig. 2).

**Table 1:** Correlation of the CEUS parameters obtained in the best perfusion area with the Johnson score and area under ROC

	PI	AUC	PIT	AUCT	TTP	AT
Correlation coefficient(r)	0.488	0.314	0.394	0.212	−0.651	− 0.597
P	0.006	0.09	0.031	0.261	< 0.001	< 0.001
Area under ROC	0.746	0.617	0.632	0.569	0.796	0.768

## Discussion

It was established that isolated regions of spermatogenic tissue may exist in the testes of men with NOA. M-TESE provides an optimal technique for sperm extraction with the guidance of an operating microscope to observe larger and opaquer seminiferous tubules. These seminiferous tubules are most likely to have ongoing spermatogenesis. In addition, M-TESE could prevent the main testicular blood supply from being damaged. Nevertheless, SRR is unsuccessful in many patients with NOA. Spermatogenesis is not distributed equally throughout the testis. Studies of patients with NOA showed that sperm quality was highest in areas with high tissue perfusion [11]. Therefore, locating areas with high perfusion would be helpful for sperm retrieval. Colour Doppler ultrasound, power Doppler ultrasound, laser flowmetry, and magnetic resonance spectroscopy have been used to locate the foci of spermatogenesis in previous studies [18]. However, these methods could only detect the main arteries in the testes, and the microvasculature was not able to be imaged. However, the high-density microvasculature greatly contributed to the high blood testicular perfusion, which was correlated with focal spermatogenesis [8, 9].

The microbubble agent used in this method (SonoVue™) is an intravascular agent and therefore does not “leak” into surrounding tissue, which provides a true representation of the microvascular supply to the testicle area. With the CEUS method, we were able to locate the high perfusion area where a rich amount of microvasculature exists before M-TESE. In our results, the SRR of the best perfusion area over the maximal longitudinal section was higher than those the poorest perfusion area and equatorial area. The best perfusion area had the densest microvasculature in the whole testicle, so the blood supply was rich in this area. It has been demonstrated that the foci of spermatogenesis might be found in regions with improved blood supply. Therefore, M-TESE performed in the best perfusion area had the best chance of detecting normal sperm. In our study, the SRR in the best perfusion area over the maximal longitudinal section was the highest, 63.1%. The CEUS group had a higher SRR than the control group. Herwig et al. [11] used a laser Doppler flowmeter and colour Doppler ultrasound to measure testicle tissue perfusion before TESE, and they found that sperm quality and quantity were highest in areas of high tissue perfusion. This conclusion was similar to ours. However, in Ullrich’s study, no significant accumulation of sperm-positive samples could be found in the main testicular vessels [18]. This conclusion did not conflict with ours, and the high perfusion area was not mostly near the main vessels, but instead occurred at positions with rich microvasculature. During M-TESE, an operating microscope cannot be used to identify the microvasculature, but CEUS could provide a new approach to image the testicle microvasculature.

In the best perfusion area from the maximal longitudinal section on CEUS of 187 testicles, we obtained pathological Johnson scores. Although sperm quality was high in areas of high tissue perfusion [11], not all of the CEUS parameters had a significant correlation with the Johnson score. In our results, PI, AT and TTP showed a relatively high correlation with the Johnson score. Pinggera et al. [10] investigated the value of the resistive index (RI) of intratesticular arteries as an indicator for normal sperm retrieval, and they found that a low RI might suggest a normal sperm count in andrological patients. When blood flows through a vessel, a lower resistance could increase blood flow. A large microvasculature and low RI may cause a high PI and low AT and TTP. According to the ROC curve, we obtained the cut-off values of the quantitative parameters to make them better predictors of successful sperm retrieval. Simultaneously, we also knew the suggested location of positive spermatogenesis. Testicles during CEUS were kept at the same position as during M-TESE. According to the measurements of the best perfusion area location over the maximal longitudinal section, the M-TESE operator could accurately find this area. With this approach, the SRR could be increased.

However, there are some limitations to our study. The best perfusion area was selected from images of the maximal longitudinal section for the testicle, so this area provided the best perfusion in one image, if not in the whole testicle. We hope additional studies will be able to use 3-Dimensional CEUS to study the whole testicle. Another limitation is the heterogenous patient population. The next research steps will include controls for age, testicular volume, and other pre-op values, such as AZFc mutations, among other factors.

## Conclusions

CEUS is able to show the microvascular distribution in testicles. The location of the best perfusion area over the maximal longitudinal section is able to guide M-TESE to improve the success rate. By using CEUS guided M-TESE, the SRR can be significantly increased. From our results, we propose that all patients with NOA receive testicle CEUS. If AT≤27 s, TTP ≤ 45 s or PI≥11 dB are found in the best perfusion area, M-TESE in this area is strongly recommended.

## Abbreviations

AT: The arrival time
AUC: Area under the curve
AUCT: The AUC of the whole testicle
CEUS: Contrast-enhanced ultrasound
ICSI: Intracytoplasmic sperm injection
M-TESE: Microdissection testicular sperm extraction
NOA: Non-obstructive azoospermia
PI: The peak intensity
PIT: The PI of the whole testicle
ROIs: The regions of interest
SRR: Sperm retrieval rate
TICs: The time-intensity curves
TTP: The time-to-peak intensity
